# Therapeutic Doses of Multipotent Stromal Cells from Minimal Adipose Tissue

**DOI:** 10.1007/s12015-014-9508-1

**Published:** 2014-05-22

**Authors:** Nan Zhang, Marilyn A. Dietrich, Mandi J. Lopez

**Affiliations:** 1 Laboratory for Equine and Comparative Orthopedic Research, Equine Health Studies Program, Department of Veterinary Clinical Sciences, Louisiana State University, Baton Rouge, LA 70803 USA; 2 Department of Pathobiological Sciences, School of Veterinary Medicine, Louisiana State University, Baton Rouge, LA 70803 USA

**Keywords:** Adipose, Multipotent stromal cell, Feline, Epididymis, Osteogenesis, Adipogenesis, Chondrogenesis, Cell isolation

## Abstract

Low yield of adult adipose-derived multipotent stromal cells (ASC) can limit autologous cell therapy in individuals with minimal adipose tissue. In this study, ASC isolation was optimized from approximately 0.2 g of feline epididymal adipose tissue for a treatment dose of 10^6^–10^7^ ASCs/kg. The ASC yield was determined for three digestions, 0.1 % collagenase in medium for 30 min (Classic), 0.3 % collagenase in buffer for 30 min (New) and 0.3 % collagenase in buffer for 1 h (Hour). After isolation by the new tissue digestion, continuously cultured ASCs (fresh) and cells recovered and expanded after cryostorage at P0 (revitalized) were characterized up to cell passage (P) 5. Outcomes included CD9, CD29, CD44, CD90 and CD105 expression, cell doublings and doubling times, fibroblastic, adipogenic and osteogenic colony forming unit (CFU) frequency percentages and lineage-specific target gene expression after induction. The New digestion had the highest CFU yield, and about 7x10^6^ ASCs/kg were available within three cell passages (P2). Compared to earlier passages, target surface antigen expression was lowest in fresh P5 cells, and fresh and revitalized P3–5 cells had slower expansion. Fresh and revitalized P1 ASCs had higher CFU frequency percentages and lineage-specific gene expression than P3. The New method described in this study was most efficient for feline epididymal ASC isolation and did not alter in vitro cell behavior. Fresh and revitalized P0-P2 feline ASCs may be most effective for preclinical and clinical trials. This study offers a potential option for ASC isolation from limited adipose tissue resources across species.

## Introduction

Adipose-derived multipotent stromal cells (ASCs) are an appealing cell option for numerous regenerative medicine therapies due, in part, to culture expansion, multipotentiality, immune privilege, trophic effects and higher in vitro proliferation compared to undifferentiated cells from bone marrow [1–3]. While ASCs can be isolated from both white (WAT) and brown (BAT) adipose tissue, WAT reportedly contains more stromal vascular cells with a greater differentiation potential [4–6]. Epididymal adipose tissue is one of the purest sources of WAT [7]. According to the 2011–2012 American Pet Products Association survey, 88 % of privately owned cats in the United States are spayed or neutered. Hence, feline epididymal adipose tissue is a promising source of WAT ASCs for feline therapeutic applications and intra-species comparisons.

Existing reports support the potential therapeutic value of feline MSCs, including neurogenic and cardiogenic capabilities of adult bone marrow-derived multipotent stromal cells (BMSCs)[8–11] and treatment of chronic kidney disease by intra-renal injection of BMSCs and ASCs [12]. About 0.2–2 % of cats in the United States suffer from diabetes mellitus, lymphoma or retinal disease [13–16]which also affect considerable numbers of other species, including humans [17–19]. There are reports of cell-based therapies for these conditions in humans and animals [17–21], but, to date, information surrounding comparable therapies for the conditions in cats is scarce.

Quality and quantity of adipose tissue varies between sites within and among individuals, human and animal [22,23]. Recent evidence also supports that orthotopic ASCs have the greatest potential for site-specific tissue regeneration, some of which have limited adipose tissue [24]. Isolation efficiency is highly variable in both humans and animals [25,26]. The ASC frequency in human subcutaneous adipose tissue cell isolates is reported to range from 0.1 to 30 % [27–29] of the cell yield, about 3 × 10^6^ ASCs/ml [29] and 0.7–3 × 10^6^ ASCs/g [30–32]. Reported therapeutic MSC doses range from 10^5^ to 10^8^ cells/kg [12,21,33–37]. The highest ASC multipotentiality and expansion rate occur up to about 10–20 cell doublings, and senescence begins at 25 to 30 cell doublings, often corresponding to about cell passage (P) 4 [38–40]. According to results from other species, 0.5 ~ 2 × 10^6^ nucleated cells can be expanded from one gram of inguinal or epididymal adipose tissue with a fibroblastic colony forming unit frequency-fibroblastic (CFU-F) percentage of 1 to 10 % [29,41–43]. Hence, for most applications, adipose tissue from individual cats must yield sufficient ASCs to provide a dose of 10^5^–10^8^ ASCs/kg within 3 to 4 cell passages (P2–3).

Prior to preclinical or clinical testing, it is imperative to fully characterize ASC populations to meet standards established by the International Society of Cell Therapy and International Federation for Adipose Therapeutics and Science that include cell phenotyping and confirmation of trilineage differentiation by gene or protein expression [44,45]. Lack of available antibodies and differences in cell behavior among species require continued efforts to meet these criteria in non-rodent animals [46]. Additionally, species-specific information about ASC isolation and culture expansion supports repeatable results with the greatest potential value for comparison among species [47]. This study addresses these necessities by optimizing feline ASC isolation and expansion to test the hypothesis that 10^6^–10^7^ ASCs/kg are available from individual cat epididymal adipose tissue within 3 cell passages before and after ASC cryopreservation.

## Materials and methods

### Study design

Epididymal tissue was harvested during routine castration of male domestic short hair cats (1.0 ± 0.2 years, 2.9 ± 0.3 kg, mean ± SEM) with a body condition score of 4–6 (range 1–9) [48]. For purposes of this study, the stromal vascular fraction (SVF) is the primary cell isolate, and P0 is the first cell passage. Initially, tissues from five cats were used to determine the ASC yield of three different tissue digestion methods: 0.1 % collagenase in Dulbecco’s modified Eagle’s medium (DMEM, Hyclone, Logan, Utah, USA) for 30 min (Classic) and 0.3 % collagenase in Kreb’s Ringer buffer for 30 min (New) or 1 h (Hour). Using the New method of tissue digestion, cell expansion rates, multipotentiality and surface marker expression were evaluated for paired fresh and revitalized (cryopreserved) cells as follows: cell percentages expressing CD9, CD29, CD44, CD90 or CD105 (P0, 1, 3, 5, fresh; P1, 3, revitalized); cell doublings (CD) and doubling times (DT) (P0-5, fresh; P1-P5, revitalized); CFU -fibroblastic (F), adipocytic (Ad) and osteoblastic (Ob) frequency percentages (SVF, P0, 1, 3, 5, fresh; P1, 3, revitalized); and lineage specific target gene expression with and without differentiation media (adipogenic, osteogenic and chondrogenic, P1, 3; fresh and revitalized). Additionally, morphology of fresh cells (SVF, P0, 1 and 3) was evaluated. To assess effects of cryopreservation, pairs of fresh and revitalized ASCs harvested from the same cats were used within assays. All assays were performed in duplicate. Stromal vascular CFU-F frequency percentages were determined from pooled tissue harvested from 10 additional, unrelated male domestic short hair cats to obtain adequate cell numbers.

## Materials

### ASC Isolation

Adipose tissue was isolated with sharp dissection, minced, and digested in type I collagenase (33 ml/g adipose tissue, Worthington Biochemical, Lakewood, NJ) within 4 h of harvest. Tissues from each cat (*n* = 5) were divided into three equal portions, and each portion was digested by one of three methods: 1) 0.1 % type I collagenase in DMEM, 0.5 h, 60 rpm (Classic); 2) 0.3 % type I collagenase in Kreb’s Ringer buffer (KRB), 0.5 h, 1,000 rpm stirring (New); and 3) 0.3 % type I collagenase in KRB, 1 h, 1,000 rpm stirring (Hour). Tissue was added to collagenase solution in a 30 ml glass jar and stirred with a stir bar at 37 °C for the New and Hour digestion methods. For the Classic digestion method, digestion mixtures within glass jars were agitated on a three-dimensional plate shaker at 37 °C. Digests were filtered and then centrifuged (260 g, 5 min). Resulting SVF pellets were resuspended in 5 ml red cell lysis buffer (0.16 mol/L NH_4_Cl, 0.01 mol/L KHCO_3_, 0.01 % EDTA) followed by cell seeding in 10 mm cell culture dishes in stromal medium (DMEM-Ham’s F12, 10 % fetal bovine serum (FBS, Hyclone), 1 % antibiotic/antimycotic solution). Medium was refreshed after overnight incubation under standard conditions (37 °C, 5 % CO_2_) and then every 2–3 days. The total number of colonies with 20 or more cells was determined after 7 days of culture. MSC density was then calculated as colony number/adipose weight (g). The New method was selected to isolate ASCs for the remainder of the study based on the highest number of colonies/g tissue. When SVF cells reached 80 % confluence, they were detached with 0.25 % trypsin and 0.1 % EDTA. Cells were seeded at a density of 5 × 10^3^ cells/cm^2^ for P0 and all subsequent passages for evaluation of fresh cells. To prepare revitalized cells, aliquots (5 × 10^5^ cells) of P0 cells were frozen in cryopreservation medium (80 % FBS, 10%DMEM, 10 % dimethyl sulfoxide) in liquid nitrogen for 30 days. Cells were then revitalized and seeded at a density of 5 × 10^3^ cells/cm^2^ in stromal medium. Revitalized cells were subsequently cultured and evaluated identically to fresh cells.

### Immunophenotype

Cell aliquots (10^5^–10^6^ cells) were resuspended in 200 μl phosphate buffered saline (PBS) containing 5 μl of antibodies against feline antigens or validated for feline cross reactivity (Table 1) and incubated for 30 min at room temperature (RT). The cells were then washed with PBS and fixed with 4 % paraformaldehyde (PFA). For CD9, CD29, CD44 and CD90, indirect immunofluorescence was performed with goat anti-mouse IgG-FITC. Cells without antibodies were used as negative autofluorescence control samples. Cellular fluorescence was evaluated by flow cytometry (FACSCalibur and CellQuest software, BD Biosciences, San Jose, California, USA). Cell percentages expressing each antigen were determined by subtracting autofluorescence control from population gated fluorescence.

**Table 1:** Fluorescence-activated cell sorting (FACS) antibodies

Antibody	Label	Target Species	Manufacturer	Cat No.
CD9	N/A	Cat	Serotec	MCA1345
CD29	N/A	Human	BD Biosciences	610468
CD44	N/A	Cat	VMRD	BAG40A
CD90	N/A	Human	eBiosciences	14–0909–80
CD105	PE	Human	eBiosciences	12–1057–41
IgG	FITC	Mouse	Sigma-Aldrich	F9006

### Cell Expansion

P0 cells were seeded in 12-well plates and counted after 2, 4, and 6 days of culture. Cell doublings and DT were determined with duplicate cultures and calculated with the formulae: CD = ln(N_f_/N_i_)/ln(2); DT = CT/CD (N_i_: initial cell number; N_f_: final cell number; CT = culture time) [49]. Day 2 and day 4 cell counts were used as the initial number to calculate day 4 and 6 expansion rates, respectively.

### Cell Morphology

Cells were cultured on glass coverslips in stromal medium for 7 days. They were washed with PBS, fixed in 4 % PFA for 20 min, and permeabilized with 0.5 % Triton-×100 for 20 min. Cell cytoskeleton was stained with Acti-stain™ 488 (1:150; Cytoskeleton, Denver, CO) and nucleus with Hoechst dye (1:2000). Slides were viewed with a fluorescent microscope (DM4500B, Leica, Buffalo Grove, IL).

### Trilineage Differentiation

After culture in stromal medium to 80 % confluence, cells were transferred to adipogenic medium (minimum essential medium alpha [α-MEM], 10 % rabbit serum, 10 % FBS, 10nM dexamethasone, 5 μg/mL insulin, 50 μM 5,8,11,14-eicosatetraynoic acid [ETYA, Cayman, Ann Arbor, MI], 100 μM indomethacin) for 10 days. They were then fixed with 4 % PFA and stained with oil red O. Similarly, cells were cultured in osteogenic medium (DMEM, 10 % FBS, 100 nM dexamethasone, 0.25 mM L-ascorbic acid) for 10 days and then in osteogenic medium supplemented with 10nM β-glycerophosphate for another 10 days. They were fixed in 70 % ethanol and stained with 2 % alizarin red. For chondrogenesis, 5 × 10^5^ cells were centrifuged (200 × g, 5 min, 15 ml tubes) to form a pellet. Pellets were cultured in chondrogenic medium (low glucose DMEM-Ham’s F12, 3 % FBS, 1 % antibiotic/antimycotic solution, 50 μg/mL ascorbate phosphate, 100 nM dexamethasone, 40 μg/mL proline, 1 mM sodium pyruvate, 1 % ITS, 10 ng/mL bone morphogenetic protein−6, 10 ng/mL transforming growth factor- β1) for 21 days. At the end of the culture period, pellets were formalin-fixed, paraffin-embedded, sectioned (5 μm) and stained with alcian blue. Parallel cultures in stromal medium were also performed to evaluate spontaneous differentiation.

### Frequency of Multipotentiality

Limiting-dilution assays to determine CFU-F, -Ad and -Ob frequency percentages were performed as previously reported with changes indicated [38]. Due to low SVF ASC numbers, samples were pooled from 10 unrelated cats, and 8 replicates of 500, 250, 125, 63, 31 and 16 cells/well were seeded for CFU-F. For all other passages, 8 replicates of 5 × 10^3^, 2.5 × 10^3^, 1.25 × 10^3^, 6.25 × 10^2^, 3.12 × 10^2^ or 1.56 × 10^2^ cells/well were seeded in 96-well plates and cultured in stromal medium for 10 days. The CFU-F colonies were fixed with 4 % PFA and stained with 0.1 % toluidine blue. For CFU-Ad and -Ob, cells were cultured in lineage-specific differentiation media and stained as described above. Wells were considered positive when there were ≥10 toluidine blue-stained colonies (CFU-F), ≥10 oil red O-stained colonies (CFU-Ad), or ≥1 alizarin red-stained colonies (CFU-Ob). The CFU frequencies were calculated as F = e^-x^ (F: ratio of negative to total wells within a row, e: natural logarithm constant 2.71, x: CFU per well). Based on Poisson’s distribution, F = 0.37 occurs when the number of total cells plated (N) in a well contains a single CFU. The CFU frequency percentage is reported as 100/N %.

### Target Gene Expression

Total RNA was extracted from cells using TRIzol reagent according to the manufacturer’s instructions. Quantity was determined with a spectrophotometer (ND-100 spectrophotometer, NanoDrop Technologies, Wilmington, DE), and 1 μg of tRNA was used for cDNA synthesis per 20 μl reaction (QuantiTect Reverse Transcription Kit, Qiagen, Hilden, Germany). Feline-specific primers were used for all reactions (Table 2), which were performed in duplicate using SYBR Green (QuantiTect) and a real-time polymerase chain reaction detection system (7900 Real-Time PCR Detection System, Applied Biosystems, Carlsbad, CA). The target gene fold change (2^−ΔΔCt^) of cells cultured in induction media was determined relative to parallel cells cultured in stromal medium using glyceraldehyde-3-phosphate dehydrogenase (GAPDH) as the reference gene.

**Table 2:** RT-PCR primer sequences

Lineage	Gene	Forward Primer (5’ – 3’)	Reverse Primer (5’ – 3’)
Reference	GAPDH	GGTCATCCCAGAGCTGAATG	AGCTTGACAAAGTGGTCATTG
Adipogenesis	PPAR-γ	GGGAGTTTCTAAAGAGCCTGAG	GTCCTCAATGGGCTTCACATTCAGC
	Leptin	CCATCTTGGACAAACTCAGGAC	GTTGAAGCTGTGCCAATCCG
Osteogenesis	Col1α1	GACAAGGGTGAGACAGGCGAACAG	CTTCTCTTGAGGTGGCTGGGGC
	OPG	GTCTCATTCGAGAAGAACCC	CACAACCGCGTGTGCGAGTGC
Chondrogenesis	Aggrecan	GCAGTACACATCATAGGTCTC	CATCGTGTTCCATTACAGAGC
	Col2α1	CACAGATTATGTCGTCGCAGAGGAC	CCAGACGCTGGTGCTGCTGACGC
Multipotentiality	Sox2	GGAGGTACATGCTGATCATG	CAGTACAACTCCATGACC
	Nanog	TTTGCTGTAACTGTATCTGGG	CCAGGCTTCTATTCCTATCACCAG

PPAR-γ = peroxisome proliferator-activated receptor gamma; Col1 = collagen type Iα1; OPG = osteoprotegerin; Col2α1 = collagen type IIα1; Sox2 = sex determining region Y-box 2; Nanog = homeobox protein NANOG

### Statistical Analysis

Results are presented as mean ± SEM. ANOVA models and Turkey’s post hoc tests were performed to evaluate the effects of isolation method on cell density (one-way) and cell passage (P0–5) and preservation (fresh or revitalized; two-way) on cell immunophenotype percentages, CD, DT, CFU frequency percentages and mRNA levels (*P* < 0.05, SAS v9.3, SAS Institute, Cary, NC). Variation between individual cats was included as the error term for analysis of fixed effects.

## Results

For all outcome measures, reported results are significant unless otherwise indicated.

### ASC Isolation

**Fig. 1:**
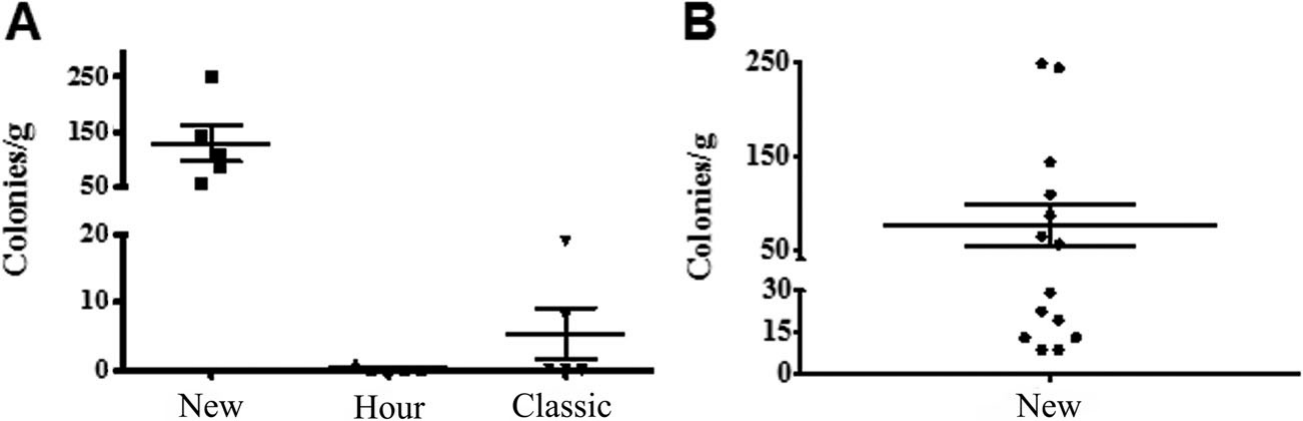
Feline epididymal adipose tissue ASC yield (mean ± SEM, *n* = 5) from three different digestion techniques (A). ASC yield (*n* = 14) from feline epididymal adipose tissue using the New digestion (B)

A mean of 0.2 ± 0.03 g adipose tissue was harvested from each cat. With tissues from 5 cats, the Hour and Classic isolation methods yielded 3.8 and 1.6 colonies/g, respectively, which were not significantly different. The New isolation method had a higher yield of 126.3 ± 33.9 colonies/g (Fig. 1). From tissues of 14 cats, the mean ASC yield was 76.3 ± 22.2 colonies/g, which was not significantly different from that with 5 cats. At 80 % confluence, there were about 1 × 10^6^ cells per 10 cm culture dish for P0-2.

### Immunophenotype

**Fig. 2:**
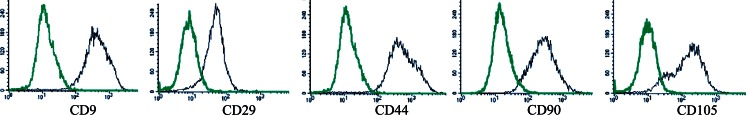
Representative immunophenotype of P0 ASCs isolated from feline epididymal adipose tissue using the New tissue digestion method. The black graphs represent stained cells and the green graphs autofluorescence

**Fig. 3:**
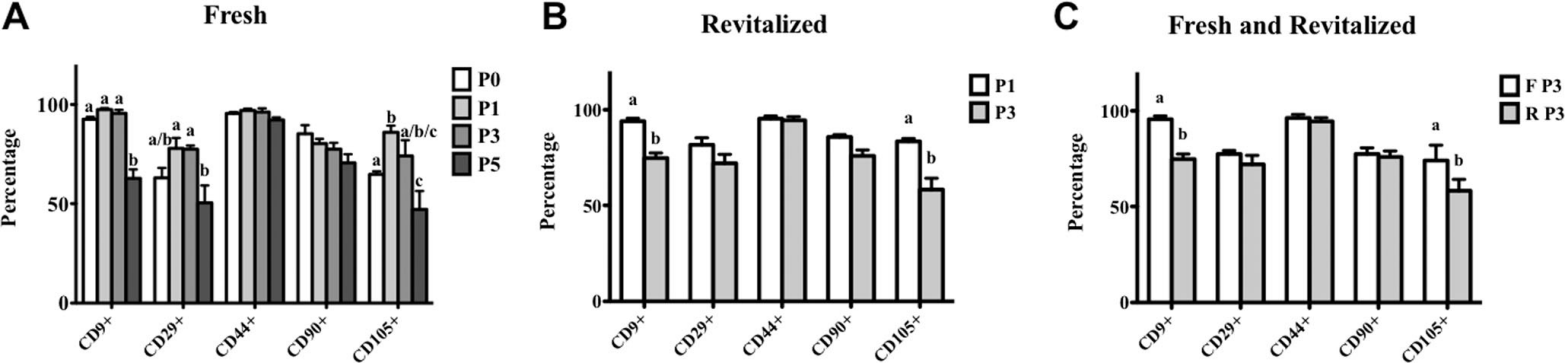
Cell percentages (mean ± SEM) of P0, 1, 3 and 5 fresh **a**, P1 and 3 revitalized **b**, and P1 and 3 fresh and revitalized **c** ASCs positive for CD9, CD29, CD44, CD90 or CD105. Columns with different letters within markers are significantly different from each other (*P* < 0.05)

The majority of P0, 1, 3, and 5 cells isolated with the New tissue digestion method were CD9+, CD29+, CD44+, CD90+ and CD105+ (Fig. 2). Among passages, fresh cell P0 had fewer CD105+ cells than P1, while P5 had fewer CD9+, CD29+ and CD105+ cells than the majority of earlier passages (Fig. 3). Revitalized P1 had more CD9+ and CD105+ cells than P3, and fresh P3 had more CD9+ and CD105+ cells than revitalized P3.

### Cell Expansion

The overall CD for P0-5 fresh cells was 0.7 ± 0.1 CD/days, and the DT was 2.8 ± 0.2 days/CD. Overall, cell expansion rates tended to decrease as passages increased (Tables 3 and 4). For fresh cells, P0 had higher CD than P1-5, and P1-2 had higher CD than P3-5 (Table 3). Cell passages P0-3 had lower DT than P4 and 5 (Table 4). For revitalized cells, P1 and 2 had higher CD than P3-5, P3 cells had higher CD than P4-5 (Table 3), P1-3 cells had lower DT than P4 and 5, and P4 had lower DT than P5 (Table 4). Fresh P4 cells had lower DT than revitalized cells, and fresh P5 cells had higher CD and lower DT than revitalized cells.

**Table 3:** Cell doublings (CDs/day) for fresh and revitalized feline epididymal ASCs isolated by the New digestion method (mean ± SEM)

	P0	P1	P2	P3	P4	P5
Fresh	1.3 ± 0.1^a^	1.1 ± 0.1^b^	0.9 ± 0.1^b^	0.5 ± 0.04^c^	0.3 ± 0.02^c^	0.3 ± 0.04^*,c^
Revitalized		1.1 ± 0.1^a^	0.9 ± 0.1^a^	0.5 ± 0.03^b^	0.2 ± 0.02^c^	0.1 ± 0.01^c^

Values with asterisks within columns are significantly different between fresh and revitalized cells. Values with different lower-case letters among passages within cell preservation (row) are significantly different (p < 0.05)

**Table 4:** Doubling times (days/CD) for fresh and revitalized feline epididymal ASCs isolated by the New tissue digestion method (mean ± SEM)

	P0	P1	P2	P3	P4	P5
Fresh	1.7 ± 0.2^a^	2.0 ± 0.2^a^	2.5 ± 0.2^a^	4.2 ± 0.4^a^	6.9 ± 0.6^*,b^	8.9 ± 1.2^*,b^
Revitalized		1.9 ± 0.1^a^	2.5 ± 0.2^a^	4.3 ± 0.4^a^	11.4 ± 1.6^b^	18.2 ± 1.2^c^

Values within columns with asterisks are significantly different between fresh and revitalized cells. Values with different lower-case letters within among passages within cell preservation (columns) are significantly different (*p* < 0.05)

### Cell Morphology

**Fig. 4:**
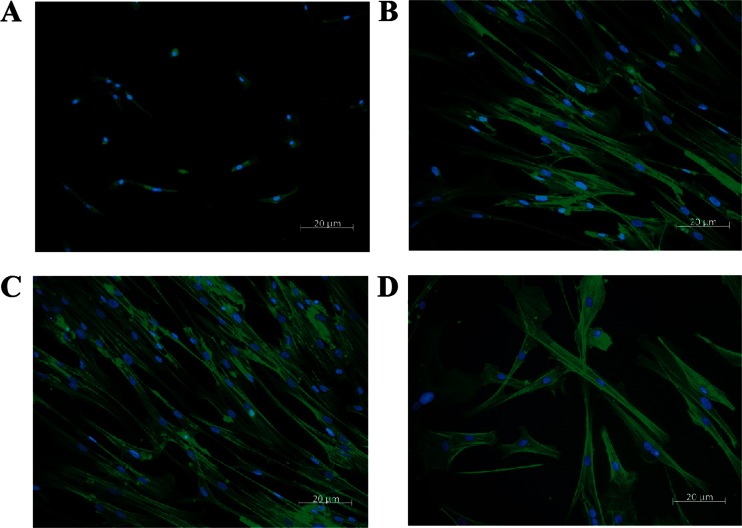
Fluorescent photomicrographs of SVF **a**, P0 **b**, P1 **c** and P3 **d** feline pididymal ASCs with cytoskeleton (actin, green) and nuclear (DNA, blue) staining (Acti-stain™ 488, actin; Hoechst dye, DNA; 20×)

Five days after seeding, the majority of adherent SVF cells had the spindle shaped morphology typical of ASCs, though small, polygonal cells were apparent (Fig. 4A). The cell population was more homogenous and organized at P0 and 1 with the same spindle shaped morphology (Figs. 4B, C). By P3, the majority of cells were polygonal and poorly organized (Fig. 4D).

### Trilineage Differentiation

**Fig. 5:**
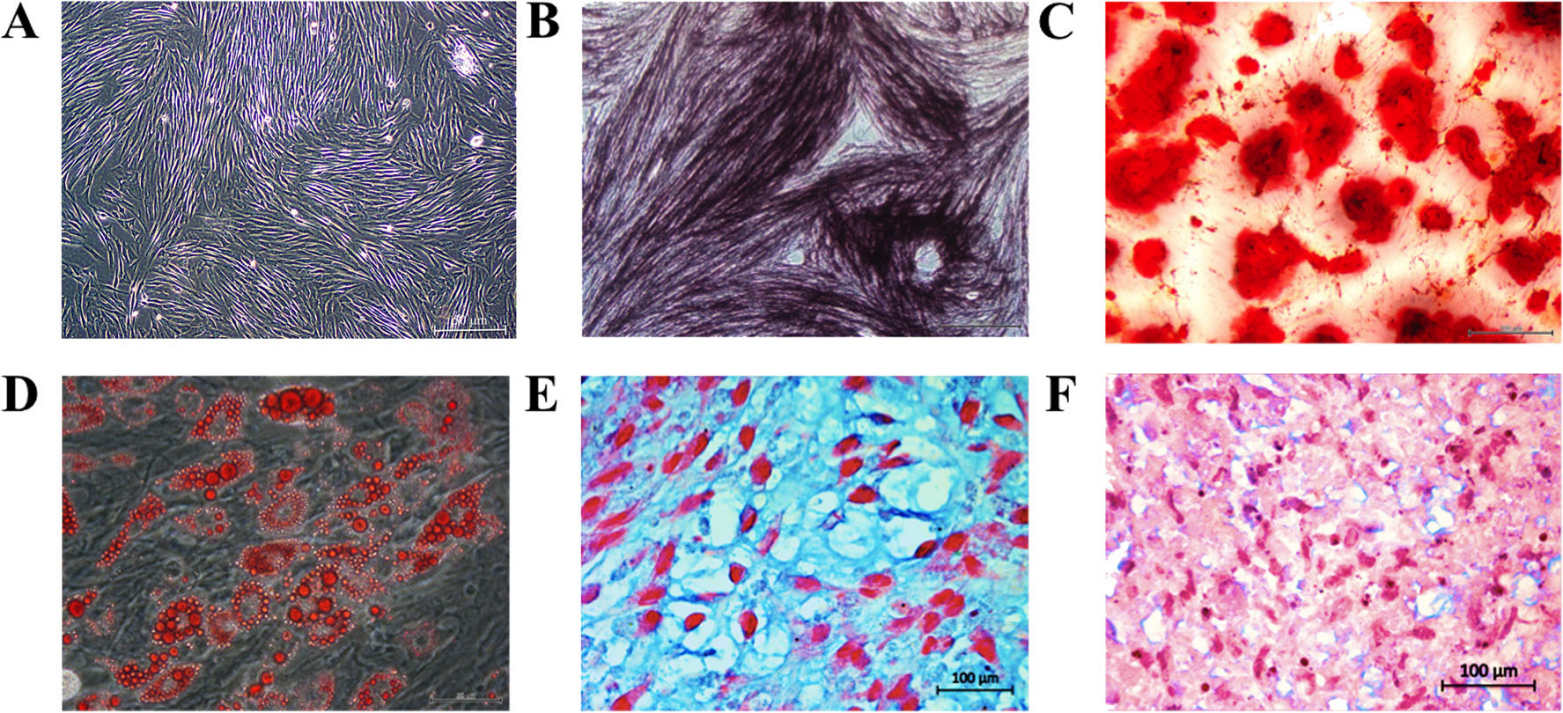
Photomicrographs of feline ASCs after 10 days of culture in stromal medium **a**, 5×; with alkaline phosphatase (ALP) staining indicating early stage osteogenesis after 10 days of culture in osteogenic medium **b**, 10×; with alizarin red staining of calcium deposition after 21 days of culture in osteogenic medium **c**, 5×; with oil red O lipid staining after 10 days of culture in adipogenic medium **d**, 40×; and showing early stage chondrogenesis based on alcian blue staining of unsulfated proteoglycans after 20 days of cell pellet culture in chondrogenic medium **e**, 64×. There is no evidence of alcian blue staining in a cell pellet cultured in stromal medium for 21 days **f**, 64×

Cells maintained a fibroblastic-like appearance when cultured in stromal media (Fig. 5A), and did not stain with oil red O, ALP or alizarin red. In osteogenic medium without β-glycerophosphate, cells expressed alkaline phosphatase (ALP) after 10 days of culture (Fig. 5B). Following the addition of β-glycerophosphate, granular nodules formed and stained with alizarin red (calcium deposition) after 21 days of culture (Fig. 5C). After about 4 days of culture in adipogenic medium, cells became round, and, after 21 days, robust lipid droplets that stained with oil red O were apparent (Fig. 5D). Cells were apparent within lacunae in pellets cultured in chondrogenic medium after 21 days, and unsulfated proteoglycans in the extra-cellular matrix stained with alcian blue (Fig. 5E). Pellets cultured in stromal medium did not have discernible lacunae or alcian blue staining (Fig. 5F).

### Frequency of Multipotentiality

The CFU frequency percentage is the percentage of cells within a population that are capable of forming CFU-F, -Ad or -Ob colonies. Due to low expansion rates after P3, revitalized P5 cells were not included in CFU assays. The SVF CFU-F frequency was 1.6 % with the New tissue digestion method (Table 5). Fresh P0 and 1 cells had higher CFU-F, −Ad, and -Ob frequencies than P3 and 5. Revitalized P1 cells had higher CFU-F, −Ad, and -Ob frequencies than P3. Also, fresh P3 cells had higher CFU-F and -Ob frequencies than revitalized cells.

**Table 5:** Colony forming unit (CFU) frequency percentages (mean ± SEM) for feline epdidymal ASCs isolated by the New tissue digestion method

Passage	Cell Preservation	CFU-F (%) (Undifferentiated)	CFU-Ad (%) (Adipogenesis)	CFU-Ob (%) (Osteogenesis)
SVF	Fresh	1.6		
0	Fresh	2.2 ± 0.2^a^	0.3 ± 0.02^a^	0.7 ± 0.2^a^
1	Fresh	2.0 ± 0.7^a^	0.3 ± 0.08^a^	0.6 ± 0.04^a^
	Revitalized	1.9 ± 0.4^a^	0.3 ± 0.03^a^	0.5 ± 0.1^a^
3	Fresh	0.1 ± 0.01^*,b^	0.03 ± 0.01^b^	0.1 ± 0.01^*,b^
	Revitalized	0.04 ± 0.01^b^	0.02 ± 0.01^b^	0.03 ± 0.03^b^
5	Fresh	0.02 ± 0.005^c^	0.002 ± 0.002^c^	0.01 ± 0.01^c^

CFU = colony forming unit; F = fibroblastic; Ad = adipocytic; Ob = osteoblastic. Values with asterisks are significantly different between fresh and revitalized cells within passage and lineage. Values with different lower-case letters among passages within lineages and preservation (fresh or revitalized) are significantly different (*p* < 0.05)

### Target Gene Expression

**Fig. 6:**
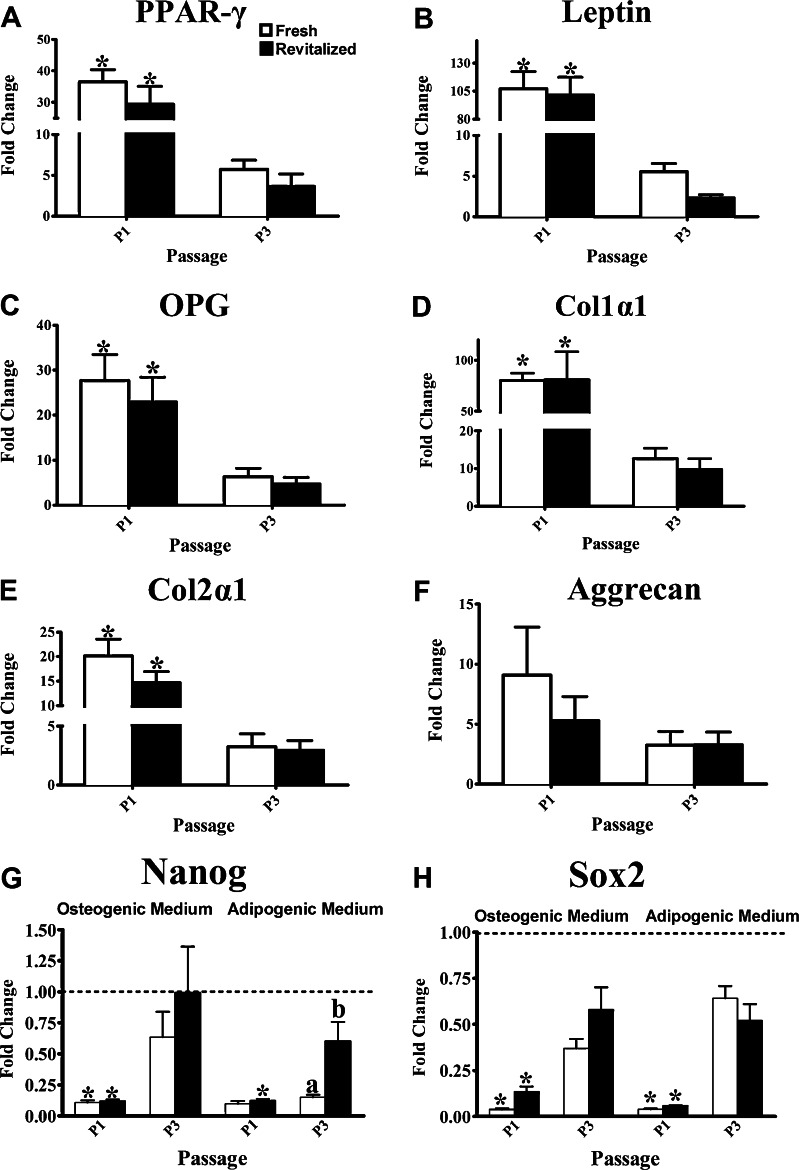
Fold change (mean ± SEM) of adipogenic (A,B), osteogenic (C,D) and chondrogenic (E,F) target gene mRNA levels in feline epididymal ASCs following culture in adipogenic, osteogenic and chondrogenic medium, respectively, relative to parallel stromal medium cultures. The fold changes in genes associated with multipotentiality (G,H) relative to stromal medium controls following culture in adipogenic and osteogenic medium are also shown. Columns lower than the dotted line are down regulated compared to stromal controls (G,H). Columns with different letters for a given passage are significantly different from each other. The P1 columns with asterisks are significantly different from P3 columns for fresh and revitalized cells (*P* < 0.05)

Compared to P3, fresh and revitalized P1 cells had higher mRNA levels of peroxisome proliferator-activated receptor gamma (PPAR-γ) and leptin following culture in adipogenic medium (Fig. 6A,B). After culture in osteogenic medium, both fresh and revitalized P1cells had higher mRNA levels of osteoprotegerin (OPG) and collagen 1α1 (col1α1) than fresh and revitalized P3, respectively (Fig. 6C,D). After culture in chondrogenic medium, both fresh and revitalized P1 cells had higher collagen 2α1 (col2α1) than fresh and revitalized P3 (Fig. 6E,F), respectively. Following culture in osteogenic medium, both fresh and revitalized P1 had lower Nanog levels than P3 (Fig. 6G). With culture in adipogenic medium, revitalized P1 had lower levels of Nanog than P3, while fresh P3 had lower Nanog levels than revitalized. Fresh and revitalized P1 had lower levels of Sox2 than P3 following culture in either adipogenic or osteogenic medium (Fig. 6H).

## Discussion

Mechanisms to generate therapeutic ASC doses from limited tissue resources will increase options for orthotopic ASC application and ASC therapies in individuals with limited adipose deposits. Toward this goal, one of three adipose digestion methods was found to yield sufficient feline ASCs from minimal tissue to give a therapeutic dose within three cell passages of fresh or cryopreserved cells, both of which retained in vitro MSC characteristics. Decreasing MSC immunophenotype, cell expansion and multipotentiality with increasing passage suggests that early passages of fresh or revitalized feline epididymal ASCs have the greatest potential for therapeutic benefit. Study results contribute to continued efforts surrounding isolation and in vitro characterization of non-rodent ASCs for comparison among species and translational applications.

Standard protocols for ASC isolation, expansion and clinical application are lacking despite promising therapeutic results in a number of species including cats [12,21,35–37,50]. Cell isolates are heterogeneous and can contain adipocyte precursors, mature endothelial cells, hematopoietic cell populations and various progenitor cells [51]. Significant variability among isolates can reduce the ability to predict post-implantation behaviour [52]. Though individual variability among animals cannot be avoided, standardized isolation and characterization techniques for specific adipose deposits may significantly enhance the predictability of ASC behaviour and efficacy following administration. The efficient, repeatable ASC isolation technique from feline epididymal adipose tissue established in this investigation is one option toward this goal.

Inconsistent cell expansion can contribute to differences among cultured ASCs since extensively expanded cells are technically “older” than those that have not been expanded as much [53]. Though SVF colony numbers were relatively low in this study, they were not significantly different among animals. Further, cell seeding density was identical for all cell passages. Adipose tissues were harvested from a relatively homogenous population of young males in this investigation. Hence, results reflect those of comparable cell populations from a specific population. Decreased CD9+ and CD105+ cell percentages in late cell passages parallel the observed reductions in multipotentiality based on CFU-Ad and -Ob frequency percentages and lineage-specific mRNA levels. Hence, these changes are unlikely to be attributable to cell isolation. Future investigations with cells isolated by the New method from different ages and/or sex will continue to expand the feline ASC knowledge base.

While the results of this study are currently limited to WAT in male cats, it is possible that they may be similar for adipose tissues accessible during other routine surgical procedures. Adipose tissue available from excised castration tissue is about a tenth of that typically harvested from other adipose depots [54]. Despite this, a “dose” of cells was available within three cell passages in this study using only plastic affinity to select for ASCs [55]. This coupled with the knowledge that cryopreserved cells maintain characteristics similar to fresh ASCs confirms the feasibility of the New isolation method to harvest cells for banking to capture the higher density and greater multipotentiality of ASCs harvested at a younger age [56–58]. The higher density of ASCs in WAT likely contributed to the observed cell yield, so the efficiency of the New digestion method will need to be independently confirmed in brown and mixed adipose tissues [5,7].

The process of cell isolation from adipose tissue is a delicate balance between adequate tissue disruption for effective cell release without damage or destruction. Adipose tissue digests typically include 0.1–0.3 % collagenase, and, for less than 0.5 g of tissue, concentrations as high as 1 %, are recommended [59]. To facilitate tissue disruption, constant shaking is usually applied during the digestion process [28]. High collagenase concentrations can be cost prohibitive and result in cell damage as can prolonged digestion times [59,60]. Alternative enzymes like trypsin tend to have lower cell yields than collagenase [60]. With a goal of optimizing cell release without high collagenase concentrations, enhanced mechanical tissue disruption by direct and indirect forces generated with a stir bar was used in this study. Also to facilitate collagenase activity, Kreb’s ringer buffer, which contains 4–5 fold fewer nutrients than DMEM, was selected for the New and Hour collagenase digestion solutions to avoid collagenous inhibition by DMEM components like glucose and sulfhydryl and chelator-containing amino acids [61–63]. The lower yield of the Hour digestion may have been a consequence of both prolonged collagenase digestion and mechanical trauma, but the more aggressive tissue disruption over a shorter period was effective in the New digestion.

The method used in this study to describe ASC yield, CFU/g, provides a direct measure with which to estimate the amount of tissue necessary for a desired cell yield. Cell yield obtained with the New method is difficult to compare to other studies since few report the number of CFU per unit tissue. The CFU/g of this study was significantly lower than the 4,590 CFU/g lapin inguinal adipose tissue previously reported [64]. A direct comparison is difficult, however, due to potential differences among digestion techniques, and CFU frequency percentage is not provided. The CFU frequency percentage of the SVF isolated by the New method was similar to previous reports in other species [28,30,31,64]. Cell immunophenotype, morphology, CD and DT and CFU-Ad and -Ob frequency percentages of cells isolated with the New digestion were similar to those reported for cats and other species [1,11,12,38,41,49,54,55]. Given the high cell yield compared to other digestion techniques, cell expansion following isolation by the New digestion method may provide greater opportunities for therapeutic doses of MSCs than other options.

Differentiation media used in this study were optimized for feline ASC based on existing knowledge from other species [11,12,38]. Adipogenic medium contained ETYA and indomethacin which promote lipid droplet formation by PPAR-γ activation, and rabbit serum, a component of canine and equine ASC adipogenic medium that contains free fatty acids for lipid production [11,65–67]. β-glycerophosphate was added to osteogenic medium after 10 days of culture to avoid colony delamination [68]. For chondrogenesis, bone morphogenetic protein-6 was added to enhance proteoglycan synthesis as reported for equine MSCs [67,69,70]. The differentiation media used in this study may be valuable for future non-rodent ASC studies, especially in the area of in vitro feline and other non-rodent mammal tissue generation.

Changes in lineage-specific gene expression was consistent with previous reports following culture in differentiation medium for fresh and revitalized cells in other species [38,54]. There were significant increases in P1 adipogenic (PPAR-γ, leptin), osteogenic (Col1α1, OPG), and chondrogenic (Col2α1) genes in corresponding differentiation media and decreases in genes associated with multipotentiality (Sox2, Nanog) following culture in osteogenesis and adipogenesis medium. Changes in P3 fresh and revitalized ASC genetic expression paralleled those of P1, but were decidedly lower. This information is consistent with the CFU frequency percentage, cell morphology and cell expansion rate findings, suggesting that ASC multipotentiality and expansion capacity decreases with passage. Current information supports greater potential for high passage bone marrow-derived multipotent stromal cells to induce instant blood mediated inflammatory reaction (IBMIR) when administered by systemic infusion versus those derived from less ex vivo expansion in humans [71]. A recent report on systemic allogeneic ASC administration for feline chronic kidney disease highlights significant adverse reactions to high doses of cryopreserved cells immediately after thawing [72]. The authors attributed the reactions to IBMIR. In light of this information and the results of this study, fresh and revitalized P0-P2 ASCs isolated by the New tissue digestion method may be most suitable for future preclinical and clinical trials.

Current research supports differences in multipotent capabilities among MSC immunophenotypes [73–76]. Direct correlations between CFU frequency percentages and cell surface marker expression are beyond the scope of this study. However, determination of the in vitro and in vivo behaviour of specific feline ASC immunophenotypes will contribute to the ability to confidently predict cell behaviour and thereby identify patients and conditions that may derive the most benefit from them. Future selection of ASCs by surface marker expression may likely increase consistency in in vitro and in vivo behaviour [52, 76].

Results from the present study demonstrate meaningful numbers of ASCs that meet cell characterization standards can be excised from limited adipose tissue in a non-rodent mammal, the cat. Characteristics were maintained after cell cryopreservation, confirming this mechanism to isolate ASCs for later application to reduce cell expansion time, avoid tissue harvest from sick or injured patients and for potential allogeneic application. Use of surface antigens and major histocompatibility markers to isolate homogenous ASC populations is a natural extension of the work reported here that may enhance cell efficacy and reduce the potential for rejection or antigenic stimulation [77] . The adipose tissue digestion method for ASC isolation developed in this study may have wide applicability for orthotopic ASC isolation and in patients with limited adipose resources.
